# Microstructure Evolution of Al6061 Alloy Made by Additive Friction Stir Deposition

**DOI:** 10.3390/ma15103676

**Published:** 2022-05-20

**Authors:** Congyuan Zeng, Hamed Ghadimi, Huan Ding, Saber Nemati, Abdelrahman Garbie, Jonathan Raush, Shengmin Guo

**Affiliations:** 1 Department of Mechanical & Industrial Engineering, Louisiana State University, Baton Rouge, LA 70803, USA; hghadi1@lsu.edu (H.G.); hding3@lsu.edu (H.D.); mnemat2@lsu.edu (S.N.); sguo2@lsu.edu (S.G.); 2 Department of Mechanical Engineering, University of Louisiana at Lafayette, Lafayette, LA 70503, USA; abdelrahman.garbie1@louisiana.edu (A.G.); jonathan.raush@louisiana.edu (J.R.)

**Keywords:** additive manufacturing, additive friction stir deposition, Al6061 alloy, microstructure, heat treatment, hardness

## Abstract

In this paper, the phase structure, composition distribution, grain morphology, and hardness of Al6061 alloy samples made with additive friction stir deposition (AFS-D) were examined. A nearly symmetrical layer-by-layer structure was observed in the cross section (vertical with respect to the fabrication-tool traversing direction) of the as-deposited Al6061 alloy samples made with a back-and-forth AFS-D strategy. Equiaxed grains were observed in the region underneath the fabrication tool, while elongated grains were seen in the “flash region” along the mass flow direction. No clear grain size variance was discovered along the AFS-D build direction except for the last deposited layer. Grains were significantly refined from the feedstock (~163.5 µm) to as-deposited Al6061 alloy parts (~8.5 µm). The hardness of the as-fabricated Al6061 alloy was lower than those of the feedstock and their heat-treated counterparts, which was ascribed to the decreased precipitate content and enlarged precipitate size.

## 1. Introduction

Metal additive manufacturing (AM) is being widely studied for its distinctive advantages, for example, substantial cost and lead-time reductions, mass reduction of components through highly efficient and lightweight designs, near-net shaping, and enhanced design freedom [1,2], for which it has gained huge interest in both the academia and industry [3]. One category of the most prevalent AM strategies is laser–powder-based additive manufacturing, including laser–powder bed fusion (L-PBF) and directed energy deposition (DED) strategies. Being high-temperature and high-energy processes [4], laser–powder-based AM strategies involve extremely fast melting and solidification processes, and the typical cooling rates for L-PBF and DED processes are 10^5^–10^7^ K/s and 10^2^–10^4^ K/s [5], respectively. Such rapid cooling rates typically lead to large amounts of residual stresses inside the AM parts and result in undesired defects such as cracks and delamination [6,7]. This demands strict requirements on the weldability of feedstock. For this reason, only limited types of metallic materials are suitable for laser–powder-based AM processes, i.e., Inconel 718 alloy [8], Inconel 625 alloy [9], stainless steel 316 L [10], Ti-6Al-4V alloy [11], and CuCrZr alloy [12]. Another undesirable feature of the laser–powder-based AM strategy is the well-known anisotropic mechanical properties caused by epitaxial solidification (columnar grains growing along the build direction), as isotropic mechanical properties are typically needed for structural applications [13]. Additionally, making spherical powders suitable for laser–powder-based AM is time-consuming and expensive, especially with specific composition requirements [13,14,15]. These drawbacks bring significant gaps between the capability of laser–powder-based AM methods and the demands of the industry.

Additive friction stir deposition (AFS-D) is a fast, scalable, and solid-state process that enables the additive manufacturing of a broad range of metals and metal matrix composites [15]. Being a thermomechanical process not going through the melting and solidification stages, the AFS-D process has little requirements on the weldability of the feedstock [16,17]. Due to the distinct manufacturing characteristics, “wrought microstructures” (fine equiaxed grains) resulting from conventional thermomechanical processes are typically observed in the AFS-D components rather than the “cast microstructures” stemming from solidification, which leads to desired isotropic mechanical properties [18,19,20]. AFS-D is a layer-by-layer fabrication process using a hollow, rotating tool to deposit feedstock material (in the form of solid rod or powders) to the substrate or previously deposited layer [21]. When the rotational tool makes contact with the substrate or previously deposited layer, severe plastic deformation occurs, and frictional heat is generated by pushing both the fabrication tool and feedstock against the substrate. Localized heat can soften the feedstock to a pasty state, which is then fed through the tool and creates metallurgical bonding with the substrate or previous layer [22]. With the motion of the fabrication tool and designed layer thickness, feedstock is deposited on the substrate layer by layer to form 3D components [13,23]. Due to the obvious advantages, AFS-D has recently been drawing the attention of more and more researchers. Rutherford et al. [20] investigated the tensile and fatigue performance of the Al6061 alloy processed with AFS-D, and it was pointed out that the as-fabricated part showed an isotropic tensile property, which was comparable with that of the annealed wrought Al6061-O alloy. Besides, it was found that the as-fabricated components exhibited similar low-cycle and high-cycle fatigue life to those of the wrought Al6061-T6 alloy. Rivera et al. [24] deposited the 2219 alloy with the AFS-D method and found that significant grain refinement occurred after material deposition, and uniform grain size was observed throughout the as-deposited cross section. Stubblefield et al. [25] accurately simulated the temperature and build profiles of the AFS-D process with a fully coupled thermo-mechanical meshfree approach on the AA6061-T651 alloy and pointed out that the temperature in the feedstock–deposition region could be up to 500 °C. A feasibility investigation on applying AFS-D to prepare Al matrix composites was also performed, and Al-SiC, A6061-Mo, and Al6061-W composites were made, showing homogeneous particle distribution without porosity [26]. In addition to Al alloys, other alloys with higher melting temperatures have also been fabricated and investigated with AFS-D processes, i.e., Inconel 625 alloy [27], Ti-6Al-4V alloy [28], stainless steel 316 L [29], and Cu 110 [30].

Despite the increasing number of studies performed on alloys made with the AFS-D strategy, previous research has mainly been focused on the relationship between the grain structures and mechanical properties of the deposition, while the detailed layer-by-layer structures and the microstructure evolution from the feedstock to the deposition are still lacking. The investigation of the layer-by-layer structures in the as-deposited parts facilitates the understanding of mass flow during the fabrication process, while the discovery of the microstructure evolution from the feedstock to the deposition helps to better understand the thermomechanical history of the manufacturing process, which is of great importance for thoroughly understanding the AFS-D process and guiding the performance optimization of the deposition. With the motivation to fill this knowledge gap, in this study, the Al6061 alloy was made with the AFS-D method, and the phase structures, composition distribution, grain structures and hardness of the feedstock, as-deposited Al6061 alloy, and heat-treated AFS-D Al6061 parts were investigated. In what follows, Section 2 demonstrates the procedures for experimentation. Section 3 describes the major results and discussion, and Section 4 presents a summary.

## 2. Experiments and Methods

Al6061 solid square rods with the composition of Al-1.63Mg-0.44Si-0.23Cu-0.16Fe (wt.%) and a size of 9.5 × 9.5 × 508 mm^3^ were used as feedstock. The surfaces of the feedstock rods were spray-coated with a thin graphite layer for lubrication purposes. A MELD L3 machine was utilized to fabricate the samples with the following processing parameters: tool rotating speed (300 RPM), tool traversing speed (254 mm/min), feedstock feed rate (152.4 mm/min). After finishing fabricating each layer, the rotational tool was lift up by 1 mm with a velocity of 30.5 mm/min. With the above fabrication parameters, 40 layers of Al6061 alloy were deposited in total. A detailed description of the AFS-D process can be found elsewhere [13,31]. The as-deposited samples were then sectioned with wire electrical discharge machining (EDM) to expose the cross sections vertical with respect to the tool traversing direction, which were the target surfaces for the microstructure, phase structure, composition distribution, and hardness characterizations. In this study, Al6061 samples with three different conditions were evaluated and compared, namely, the Al6061 feedstock, as-deposited Al6061 parts, and AFS-D Al6061 parts after heat treatment. Hereafter, the three types of samples are denoted as Feedstock, AD, and ADHT, respectively, for easy distinguishing. T6 heat treatment was performed on the as-deposited Al6061 parts (wrapped with stainless-steel foil to minimize oxidation) in a Ney Vulcan 3-550 Dental Furnace (air atmosphere) with the following two steps: (i) Solution treatment: the sample was heated up to 530 °C with a heating rate of 10 °C/min and then kept at 530 °C for 45 min, followed by quenching in room-temperature water. (ii) Artificial aging treatment: with a heating rate of 10 °C/min, the sample was heated to 175 °C, then kept at 175 °C for 8 h, and finally quenched in room-temperature water [32]. Prior to the characterization of the samples, the sample surfaces were mechanically ground with SiC papers of different grit sizes (320, 600, 800, 1000, and 1200 grits, in sequence), polished with MetaDi^TM^ Supereme polycrystalline diamond suspension (6 μm, 3 μm, and 1 μm, in sequence), followed by vibratory polishing with 50 nm silica suspension on a Pace Technologies GIGA 0900 Vibratory Polisher for 12 h. X-ray diffraction (XRD; Panalytical Empyrean) was carried out to determine the phase structures of the samples with a θ-2θ angular range of 20–90° using a scanning step size of 0.026°. Calculation of phase diagram (CALPHAD) predictions were conducted to guide the phase structure analysis of the Al6061 alloy with the commercial ThermoCalc database of TCAL6:Al Alloys v6.0. To reveal the layer-by-layer structures on the cross section of the AD sample, an etching solution containing nitric acid and hydrochloric acid with a volumetric ratio of 1:3 was used. After etching for 25 s, the cross sections were cleaned with flushing water and dried in air. A scanning electron microscope (SEM; FEI Quanta3D FEG Dual-Beam) with attachments for energy-dispersive spectroscopy (EDX) and electron backscatter diffraction (EBSD) were used for microstructural evaluation and composition distribution analysis. Vickers hardness was determined with a testing load of 100 gf and a dwell time of 15 s using a digital micro-hardness tester (Clark Instrument Model CM-802AT; Novi, MI, USA).

## 3. Results and Discussion

### 3.1. Phase Structure

**Figure 1:**
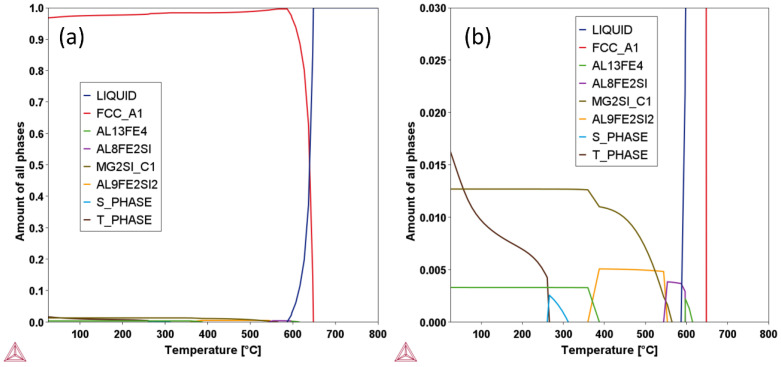
Thermo-Calc prediction results of the Al6061 alloy demonstrating the amounts of phases as a function of temperature: (**a**) whole range of the prediction; (**b**) narrow range showing the secondary phases.

Figure 1 demonstrates the amount of each phase as a function of temperature for the Al6061 alloy in the equilibrium state. According to Figure 1a, FCC-Al was the main phase of the Al6061 alloy, which was over 96.8% (mole percentage) at room temperature. By comparison, the amounts of secondary phases were nearly negligible. Figure 1b shows the narrow range of Thermo-Calc prediction for the secondary phases. Clearly, the Mg_2_Si phase, T-Phase (Al_9.4_Mg_6.2_Cu) and Al_13_Fe_4_ phase were the three secondary phases at room temperature, with a total amount of approximately 3.2%.

**Figure 2:**
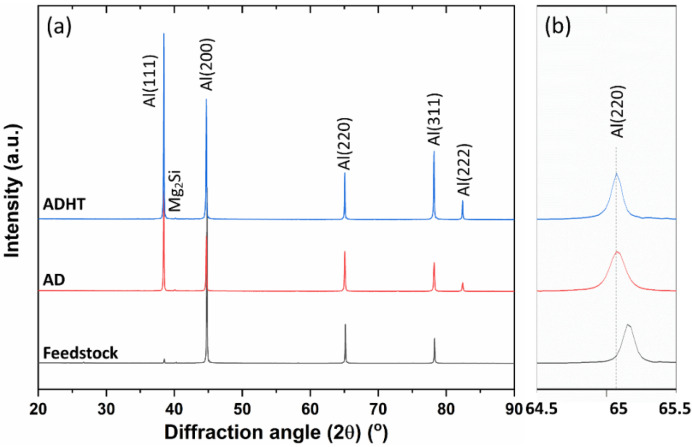
(**a**) XRD patterns of the three samples; (**b**) XRD patterns of the samples over a narrow diffraction angle range.

Figure 2 shows the XRD characterization results of the Feedstock, AD, and ADHT samples. Consistent with the Thermo-Calc prediction, the main peaks of the samples belonged to the FCC-Al phase. Following a close observation, a weak diffraction peak of the Mg_2_Si phase was also observed for all three samples around the diffraction angle of 40.2°. Figure 2b shows the diffraction peak of FCC-Al(200) within the narrow XRD diffraction angle range from 64.5° to 65.5°. Interestingly, the diffraction peaks of the AD and ADHT samples nearly overlapped each other, while that of the Feedstock shifted to the larger diffraction angle side. According to Bragg’s equation, the increase in diffraction angle indicates the decrease in interplanar spacing, equally meaning the decrease in lattice parameters. Therefore, after the AFS-D process, the lattice parameters of the AD and ADHT samples increased, which implies that the precipitates dissolved back into the Al matrix as solid solutes. A similar phenomenon has also been observed by other researchers [20,33]. The dissolution of precipitates after AFS-D processing can be explained by the temperature profile during the AFS-D process. A temperature above 250 °C would trigger the dissolution of precipitates in the Al6061 alloy [34]. Moreover, it has been reported that with an inserted thermocouple in the fabrication substrate, the temperature during the AFS-D process can reach up to 500 °C [20,25], which is sufficient to initiate the precipitate dissolution process.

### 3.2. Composition Distribution

**Figure 3:**
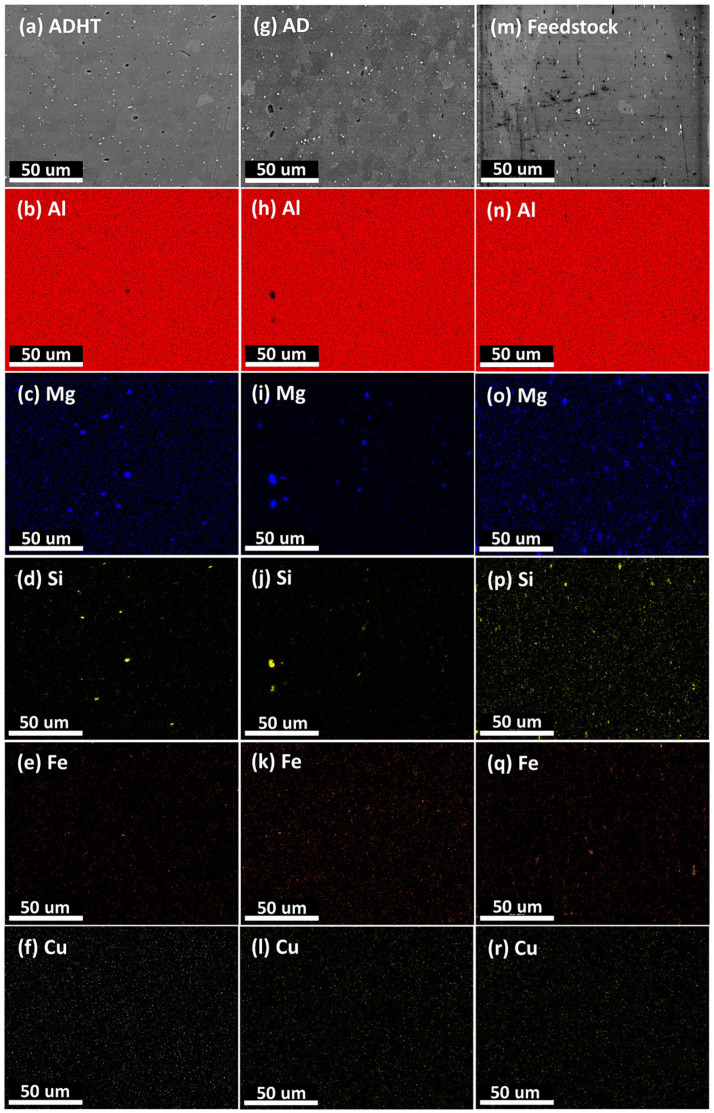
EDS mapping results showing the distribution of elements in the samples of ADHT (**a**–**f**), AD (**g**–**l**), and Feedstock (**m**–**r**).

The compositional distributions in the samples are shown in Figure 3. Based on the measurements, Al was clearly observed as the matrix element. Mg and Si were also obvious as the primary alloying elements existing as Mg_2_Si phase or T-Phase (according to Thermo-Calc prediction and XRD test results), which play a significant role in strengthening the Al6061 alloy [35,36]. Meanwhile, due to their much lower contents, Fe and Cu were hardly seen, especially the latter. By comparing the distribution of the elements Mg and Si, differences were discovered in the three samples. Precipitates of Mg and Si elements distributed uniformly in the Al matrix in the Feedstock sample (Figure 3n–p). However, in the AD sample, precipitates became larger, which is apparent in Figure 3h–j. After T6 heat treatments, precipitates became smaller again (Figure 3b–d). The variation in behavior observed in the precipitates in the samples can be explained below. AFS-D is a thermophysical process, and the frictional heat results in a temperature of the fabrication zone up to 500 °C [20,25]. During the layer-by-layer fabrication process, heat accumulates, holding an elevated temperature inside the deposited parts (acting as a dynamic heat treatment process), which causes precipitates to grow. It is worth noting that only micro-sized precipitates could be observed with the EDS mapping results in this study. Nano-sized precipitates, i.e., the *β*″ phase (Mg_5_Si_6_), also exist in Al6061 alloys, especially after T6 heat treatments [36,37,38].

### 3.3. Microstructure

**Figure 4:**
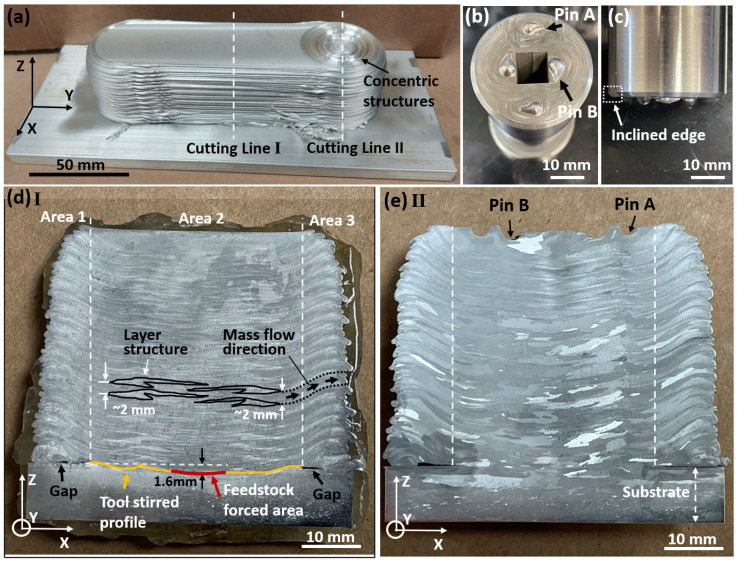
Photos illustrating the as-deposited Al6061 alloy parts (**a**), overview of the tool with pin structures (**b**), side view of the tool (**c**), cross section along Cutting Line I after etching (**d**), and cross section along Cutting Line II after etching (**e**).

AFS-D is a layer-by-layer fabrication method, which is evidenced by the repeated flash structures on the side of the as-deposited part shown in Figure 4a. This part was deposited with the back-and-forth strategy (along the *Y*-axis in Figure 4a), with the final deposition spot on the top right. After lifting the rotating tool, characteristic concentric-circle grooves were generated due to (i) the stopping of feedstock supply and (ii) the unique structure of the tool demonstrated in Figure 4b. Based on Figure 4b, it is clear that two sets of circle-symmetrical pins with different sizes and spacing with respect to the tool center were on the tool surface with water-drop shapes, which stirred the squeezed-out feedstock, introducing more severe plastic deformation. Figure 4c reveals more details of the fabrication tool from a side view. The two sets of pin structures had different heights, with the inner pins being 2.3 mm in height and the outer ones being 1.8 mm. In addition, the edge of the fabrication tool was chamfered with an angle of around 45°. Cross sections (XZ planes) vertical with respect to the tool traversing direction (along the *Y*-axis) were exposed along Cuttings Lines I and II, as shown in Figure 4a. Figure 4d shows Cross Section I along Cutting Line I before heat treatment, while Figure 4e demonstrates Cross Section II along Cutting Line II after T6 heat treatments. After polishing and etching, the layer-by-layer structures were revealed.

Based on Figure 4d, the cross section could be divided into three regions, namely, Area 1, Area 2, and Area 3. Area 2 was right underneath the tool, with the width equal to the outer diameter of the tool (38.1 mm). Due to the restriction of the tool surface, the topmost surface layer in Area 2 was flat. By comparison, materials that flowed outside of the tool (Areas 1 and 3) moved with a trend towards the building direction (*Z*-axis) first and then gradually along the *Y*-axis direction (horizontally). At the beginning of the AFS-D process, the fabrication tool and the feedstock were pushed against the substrate to initiate good metallurgical bonding between the deposited feedstock and the substrate with frictional heat. The red curve in Figure 4d stands for the feedstock-forced area, while the orange curves represent the tool-stirred profile into the substrate. The deepest penetration depth lay on the feedstock-forced area, which was around 1.6 mm. Representative etched layer structures are highlighted in Figure 4d with a hook-barb-like structure. The barb structures typically lay between Pins A and B, which most likely resulted from the mass back flow caused by the block of Pin A during the feedstock mass transport. Interestingly, the hook-barb-like structures were not symmetrical to the central line along the building direction; this, however, appeared every two fabrication layers on each side of the central line. This observation is related to the back-and-forth (*Y*-axis direction in Figure 4a) fabrication strategy. Along the layer structures, the mass flow direction from underneath the tool to Area 3 (outside of the tool) are indicated by the black arrows and the dotted curves. There are two passes within the dotted curves. The trend of the mass flow can be explained by the tool design (Figure 4c). Gaps were observed on the bottom deposited layer in Areas 1 and 3 right next to the tool (Figure 4d,e). By observing the gaps, it was deduced that when depositing the first layer, the paste-state feedstock traveling outside of the tool was led by the tool surface chamfer without contacting the substrate until a certain distance due to gravity. After the paste-state feedstock contacted the substrate again, more feedstock accumulated above the attached area, forming a protuberant surface, which acted as the substrate for the subsequent deposited layers. It is worth noting that, unlike those in Area 2, the new layers in Areas 1 and 3 were deposited on previous layers without going through severe inter-layer plastic deformation. Areas 1 and 3 could be removed in multi-pass AFS-D processes or by post AM machining.

Figure 4e displays the etched cross section along Cutting Line II (Figure 4a) after heat treatment. Similar to Cross Section I (Figure 4d), Cross Section II could also be divided into three areas. Besides, layer-by-layer structures were nearly identical for the two cross sections (Figure 4d,e). The distinctive variance was within the topmost surface layer, where Cross Section II showed the rotational-tool pin structures, with the shallow and deep ones belonging to Pin A and Pin B, respectively.

**Figure 5:**
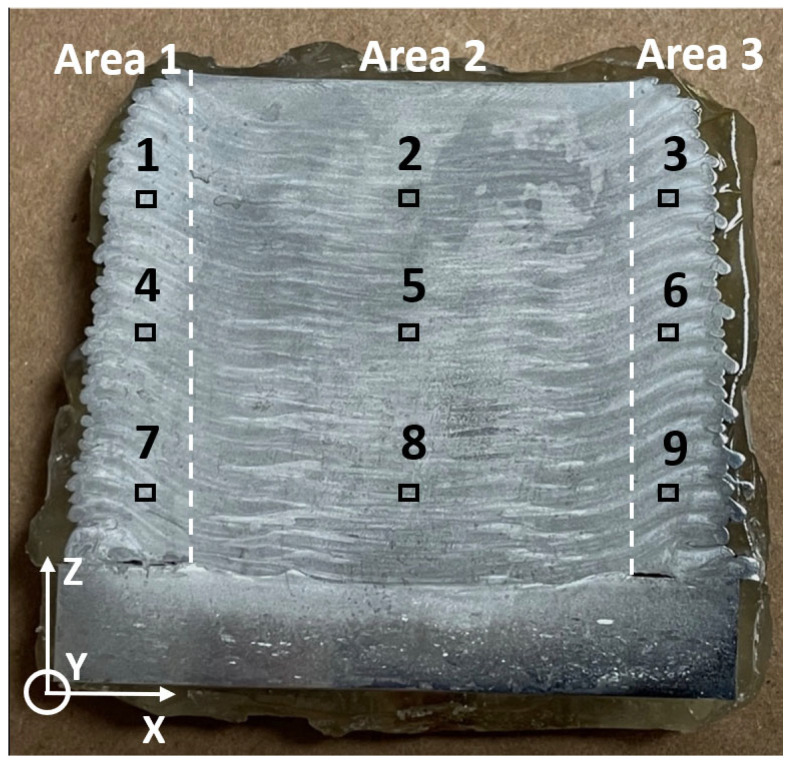
Image showing the nine spots for microstructural tests on the cross section of the samples.

Figure 5 indicates the spots for the microstructural tests on Cross Section I (Figure 4). Three spots lay in each area uniformly distributed on the same line along the build direction (*Z*-axis). Likewise, three spots from the three areas, for example, Spots 1, 2, and 3, were on the same horizontal level. The selection of the test spots aimed at evaluating the microstructural evolution along both the building direction (*Z*-axis) and horizontal direction (*X*-axis).

**Figure 6:**
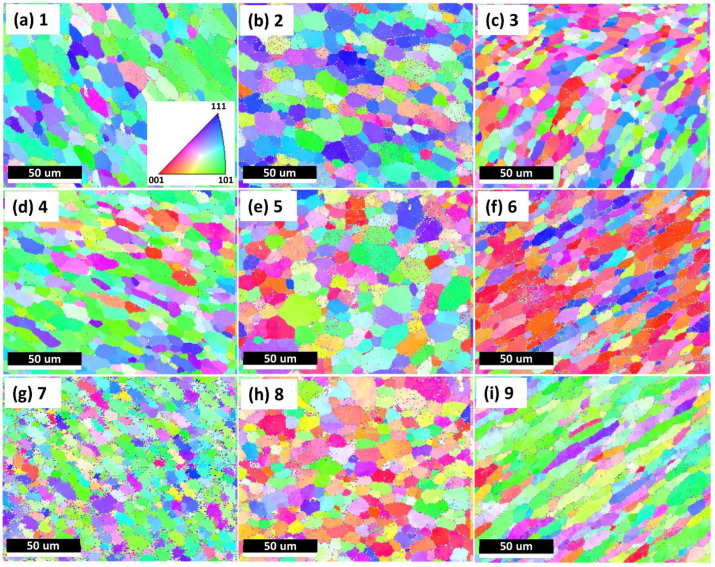
EBSD inverse pole figure (IPF) orientation maps of the as-deposited Al6061 alloy on different spots. (**a**–**i**) Numbers 1–9 are corresponding to the nine locations shown in Figure 5.

Figure 6 shows the EBSD inverse pole figure (IPF) orientation maps on the cross section shown in Figure 5. Generally in good agreement with the etched cross sections observed in Figure 4, the grain structures were symmetric to the central line of the cross section, with equiaxed grains in Area 2 and elongated grains in Area 1 and Area 3 following the mass flow direction. No significant grain-size differences were observed in the three spots for each area, indicating uniform grain size distribution along the building direction (*Z*-axis). A similar observation has also previously been reported [24]. AFS-D is a solid-state fabrication process; however, a relatively high temperature is generated and held inside the deposited part, which can be up to 500 °C in Al alloys [20,25]. Therefore, dynamic heat treatment was performed on the deposited part due to the temperature. Earlier deposited layers were kept in the high-temperature state for a prolonged time. Considering the grain size results observed above, it is reasonable to state that the grain size of the deposited part was mainly dependent on the maximum processing temperature. In real applications, components with isotropic mechanical performance are preferred [13]. Isotropic crystallographic orientations (equiaxed grains) typically lead to isotropic mechanical properties [39], while elongated grains result in anisotropic mechanical behaviors [40]. Further, unlike Area 2, only a “kissing” attachment existed between layers in Areas 1 and 3 rather than metallurgical bonding, which are called flash regions. In real applications, Area 1 and Area 3 would be removed in multi-pass AFS-D processes or by post AM machining. In the remainder of the text, only Area 2 is considered for investigation for the as-deposited sample both before and after heat treatments. The average grain size for Area 2 was measured to be 8.5 ± 3.1 µm.

**Figure 7:**
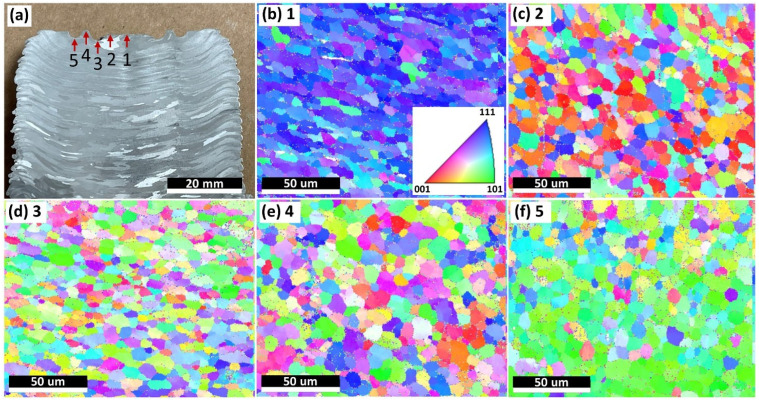
Microstructural examination of the last deposited layer. (**a**) The positions of the test spots on the sample cross section. Spot 1: center of the top layer; Spot 2: the point the mass flow started contacting the first pin; Spot 3: the region close to the top of the first pin; Spot 4: the region between the first and second pin; Spot 5: the region close to the top of the second pin. (**b**–**f**) The EBSD inverse pole figure (IPF) orientation maps of Spots 1–5, respectively.

As per Figure 6, the microstructures inside the as-deposited parts (not on the topmost surface) were investigated. According to Figure 4b,c, a tool with specially designed pin structures was used. Therefore, each deposited layer, excluding the final deposited layer, was stirred more than twice considering a layer thickness of 1 mm. It is interesting to examine the microstructural information of the final deposited (topmost) layer that was only stirred once to find out the difference with other layers stirred over two times. Figure 7 shows the microstructures of the last deposited layer in the cross section where the deposition was finished, and the tool was lifted. The microstructural evaluations were performed at five different spots in the topmost layer within 1 mm thickness, as shown in Figure 7a, which were all underneath the fabrication tool, specifically, at the center of the feedstock–deposition interaction region and around the pin-structure regions. Based on Figure 7b–f, the grains were mainly equiaxed in the spots, except for Spot 1 (center of the top layer), which was the spot not stirred by the pin structures. The grain size of the last deposited layer (7.7 ± 1.8 µm) was slightly smaller than the inner parts in Area 2 (Figure 6). This observation was most likely due to direct exposure to the environments, which showed a higher cooling efficiency, leading to slightly lower temperature in the topmost deposited layer.

**Figure 8:**
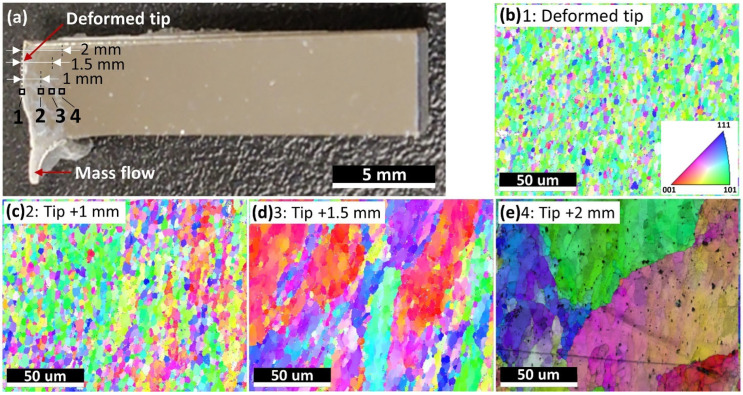
Images showing the residual feedstock and its microstructures. (**a**) A photo illustrating the shape of the quarter residual feedstock with Spots 1, 2, 3, and 4 being the microstructural characterization regions. Spot 1 was right on the deformed tip, while Spots 2, 3, and 4 were 1 mm, 1.5 mm, and 2 mm vertically away from the deformed surface. (**b**–**e**) EBSD inverse pole figure (IPF) orientation maps of Spots 1–4.

The square feedstock rods were not entirely consumed during fabrication to prevent the actuator (used for feedstock feeding) from contacting the deposited parts. Residual feedstock rods with lengths of 25–50 mm were collected. Different from the as-deposited parts remaining at relatively high temperature for a considerable time, residual feedstock went through air cooling, which tends to retain the microstructure in the regions right between the deposited parts and the feedstock. The investigation of the microstructures of the residual feedstock would give a better understanding of the microstructure evolution from the feedstock to the deposited parts during the AFS-D process. To discover the microstructure variation behavior, the residual feedstock was cut by splitting the bottom surface into four identical squares along the central axis of the feedstock with a wire EDM machine. Four identical feedstock rods were then obtained, and Figure 8a shows the image of one of them after surface polishing. According to Figure 8a, feedstock deformation was clearly observed, which reflects the mass flow during the AFS-D process. Four spots were examined for microstructural observation, namely, Spots 1–4, shown in Figure 8a. Figure 8b,e show the grain structure evolution as a function of the distance vertically away from the deformed surface. Obviously, significant grain size variation was seen. Specifically, the average grain sizes for Spots 1–4 were approximately 3.3, 4.2, 10.1, and 163.5 µm, respectively, with Spot 4 being comparable to the grain size of feedstock without deformation. Therefore, it was deduced that the feedstock started to deform around 2 mm away from the feedstock–deposition interface. It is worth noting that the grains close to the feedstock-deformed tip (i.e., Spots 1 and 2) were clearly smaller than those of the as-deposited parts. This observation illustrates the grain growth in the as-deposited part after deposition. Therefore, during the AFS-D process, due to the high shear and severe plastic deformation, the feedstock started to deform 2 mm away from the interaction surface. Moreover, due to dynamic recrystallization, caused by the high shear and severe plastic deformation, and frictional heat resulted from the interaction between the feedstock/tool and the previously deposited layer, clear grain refinement occurred as it got closer to the feedstock–deposition interface (from around 163.5 µm to 3.3 µm) [41,42]. After deposition, due to the frictional heat accumulated, the grain size of the deposited part grew to around 8 µm.

**Figure 9:**
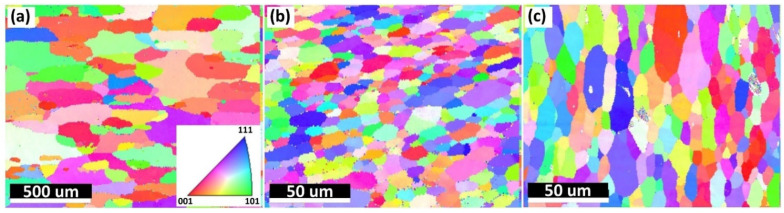
EBSD inverse pole figure (IPF) orientation maps of the Feedstock (**a**), AD sample (**b**), and ADHT sample (**c**). The EBSD image for the feedstock is at a 10-time lower magnification.

Figure 9 shows the microstructures of the Feedstock, AD, and ADHT samples. By considering the scale bar in the images, Feedstock showed a remarkably larger grain size than the other two samples, while the grain size of the ADHT sample was slightly larger than that of the AD sample. Quantitatively, the grain sizes for the Feedstock, AD, and ADHT samples were 163.5 ± 96.2 µm, 8.5 ± 3.1 µm, and 12.5 ± 5.2 µm, respectively. The grain size increase of the ADHT sample was due to the high solution treatment temperature (530 °C). Such a grain size variance should show an effect on the mechanical performance. However, it is also noteworthy that Al6061 is a precipitate-hardening alloy. According to the XRD test results in Figure 2 and the EDS mapping test data in Figure 3, the precipitates showed different sizes and distributions in the Al6061 alloys, which could also play a critical role in the mechanical behaviors.

### 3.4. Vickers Hardness

**Figure 10:**
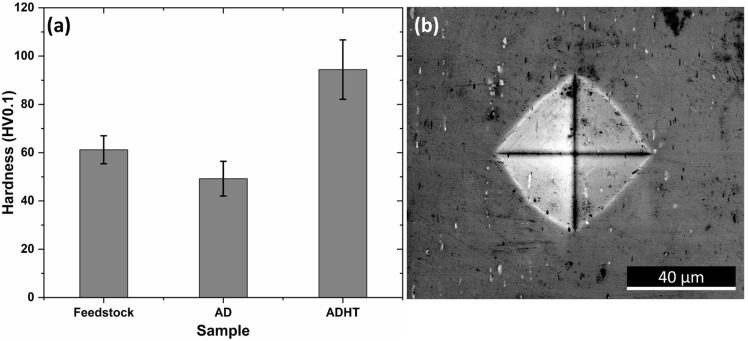
(**a**) Hardness test results of the samples; (**b**) SEM image showing the indentation of hardness test for the AD sample.

To gain an understanding of the mechanical performance of the Feedstock, AD, and ADHT samples, Vickers hardness tests were performed, and the test results are shown in Figure 10a. Clearly, the ADHT sample showed the highest hardness (94.4 ± 12.3 HV0.1), while Feedstock had the medium hardness values (61.2 ± 5.8 HV0.1), and the AD sample possessed the lowest hardness (49.2 ± 7.2 HV0.1). This observation shows a clear discrepancy with that of the grain-size results. Figure 10b shows the indentation shape and size on the surface of the AD sample after the Vickers hardness test. The diagonal length was around 57 µm, which covered multiple grains. Therefore, the Hall–Petch effect (grain boundary strengthening) was not the main strengthening mechanism for the AD and ADHT samples. As mentioned above, Al6061 is a precipitate-hardening alloy; the precipitate conditions, including precipitate sizes and amounts, show a significant effect on the mechanical performance. According to Figure 2, it was found that Feedstock contained more precipitates, while the AD and ADHT samples had lower but almost identical amounts of precipitates. In addition, according to Figure 3, the precipitates in the AD samples grew to larger sizes than their counterparts, indicated by the EDS mapping results for the elements Al, Mg, and Si. Apart from the micro-sized Mg_2_Si precipitates observed in Figure 2 and Figure 3, nano-sized precipitates existed in the Al6061 alloy, especially after the T6 heat treatments, specifically the needle-shaped *β*″ meta stable phase [43], which is the main strengthening phase for heat-treated Al6061 alloys [44]. Precipitation strengthening results from the ability of precipitates to restrain the movement of dislocations.

Compared with micro-sized precipitates (observed in Figure 3), nano-sized precipitates (*β*″ phase as reported) play the dominant role in the strengthening of the Al6061 alloy [43,44,45,46]. Moreover, it is commonly accepted that the Al6061 alloy after T6 heat treatment contains more nano-sized *β*″ phase. This is the reason why the ADHT sample showed much higher hardness values than both the Feedstock and AD samples, although the micro-sized precipitate amounts of the Feedstock sample were even higher. Similarly, due to the higher amounts of precipitates and relatively smaller sizes of precipitates, the Feedstock sample possessed higher hardness than the AD sample.

To sum up, it is clear that the AFS-D process had a significant effect on the (i) grain structures and (ii) size and distribution of the precipitates inside the deposition. Due to the high shear and severe plastic deformation, dynamic recrystallization occurred at the feedstock–deposition interface, and the grain size of the feedstock was significantly refined. After fabrication, the deposition was held at a relatively high temperature due the frictional heat accumulation, which led to slight grain growth. However, compared with the feedstock, the grain size was still remarkably reduced in the deposition. In the meantime, due to the frictional heat generated during the fabrication process, dynamic heat treatment was performed on the deposited components, which, most likely, led to the dissolution and growth of precipitates at the same time, based on the XRD and EDS mapping results. However, it is worth noting that the influence of the characteristic high shear and severe plastic deformation of the AFS-D process on the state of precipitates is still unknown and remains as future work. Although the significant refinement of grain size typically leads to mechanical strength improvement due to grain-boundary strengthening, for a precipitation hardening alloy, i.e., Al6061 in this study, the state of the precipitates inside the deposited component plays the dominant role in determining the strength. Therefore, after the AFS-D process of precipitation-hardening alloys, heat treatment is necessary to tune the size and distribution of precipitates for optimizing their mechanical performance.

## 4. Conclusions

In this paper, the phase structure, composition distribution, microstructure, and hardness of the feedstock and Al6061 alloy made with AFS-D both before and after heat treatments were evaluated. Moreover, the below conclusions were reached.

(1) In the AFS-D process, equiaxed grains were typically observed underneath the fabrication-tool region, while elongated grains were seen in the “flash region” outside of the underneath-tool region due to the mass transfer flow.
(2) During the AFS-D fabrication process, due to severe plastic deformation, the Al6061 alloy feedstock started to deform approximately 2 mm away from the feedstock–deposition contact interface. Moreover, the grain sizes of the feedstock sharply decreased from an average value of 163.6 µm to 3.3 µm on the contact surface. Because of the heat generated and accumulated during the AFS-D process, the grains grew to an average size of around 8.5 µm in the region underneath the fabrication tool. No significant grain size variance was noticed along the building direction except for the final deposited layer, which showed an average grain size of 7.7 µm.
(3) After the AFS-D process, the grain size of the Al6061 alloy was 8.5 ± 3.1 µm. After the solution + artificial aging two-step heat treatment, the grains of the Al6061 alloy made with AFS-D grew to a size of 12.5 ± 5.2 µm.
(4) The hardness of the as-deposited Al6061 alloy sample was lower than that of the feedstock and as-deposited Al6061 alloy after heat treatment, which was ascribed to the lower precipitate amount and increased precipitate size.

Future research directions include (i) the investigation of the effect of the high shear and severe plastic deformation on the state of the precipitates and (ii) the systematical mechanical performance evaluation of the deposition at different regions in the deposited components, i.e., the center or the edge regions under the fabrication tool.
